# Functionality of IAV packaging signals depends on site-specific charges within the viral nucleoprotein

**DOI:** 10.1128/jvi.01972-23

**Published:** 2024-03-12

**Authors:** Kevin Ciminski, Viktoria Flore, Celia Jakob, Helen Mues, Anne Smedegaard Frederiksen, Martin Schwemmle, Hardin Bolte, Sebastian Giese

**Affiliations:** 1 Institute of Virology, Freiburg University Medical Center, Faculty of Medicine, University of Freiburg, Freiburg, Germany; Emory University School of Medicine, Atlanta, Georgia, USA

**Keywords:** influenza A virus, genome packaging, lysine acetylation, packaging mutant, NP residues, terminal packaging signals

## Abstract

The coordinated packaging of the segmented genome of the influenza A virus (IAV) into virions is an essential step of the viral life cycle. This process is controlled by the interaction of packaging signals present in all eight viral RNA (vRNA) segments and the viral nucleoprotein (NP), which binds vRNA via a positively charged binding groove. However, mechanistic models of how the packaging signals and NP work together to coordinate genome packaging are missing. Here, we studied genome packaging in influenza A/SC35M virus mutants that carry mutated packaging signals as well as specific amino acid substitutions at the highly conserved lysine (K) residues 184 and 229 in the RNA-binding groove of NP. Because these lysines are acetylated and thus neutrally charged in infected host cells, we replaced them with glutamine to mimic the acetylated, neutrally charged state or arginine to mimic the non-acetylated, positively charged state. Our analysis shows that the coordinated packaging of eight vRNAs is influenced by (i) the charge state of the replacing amino acid and (ii) its location within the RNA-binding groove. Accordingly, we propose that lysine acetylation induces different charge states within the RNA-binding groove of NP, thereby supporting the activity of specific packaging signals during coordinated genome packaging.

**IMPORTANCE:**

Influenza A viruses (IAVs) have a segmented viral RNA (vRNA) genome encapsidated by multiple copies of the viral nucleoprotein (NP) and organized into eight distinct viral ribonucleoprotein complexes. Although genome segmentation contributes significantly to viral evolution and adaptation, it requires a highly sophisticated genome-packaging mechanism. How eight distinct genome complexes are incorporated into the virion is poorly understood, but previous research suggests an essential role for both vRNA packaging signals and highly conserved NP amino acids. By demonstrating that the packaging process is controlled by charge-dependent interactions of highly conserved lysine residues in NP and vRNA packaging signals, our study provides new insights into the sophisticated packaging mechanism of IAVs.

## INTRODUCTION

The influenza A virus (IAV) genome consists of eight distinct viral RNAs (vRNAs). Depending on their length, these vRNAs are bound by multiple copies of viral nucleoprotein (NP) and one copy of a heterotrimeric viral polymerase to form eight viral ribonucleoprotein complexes (vRNPs) (1). Genome segmentation allows IAVs to exchange gene segments through reassortment in co-infected host cells. This process occurs readily and has contributed to the emergence of several pandemic viruses in the past (2, 3). Although genome segmentation is evolutionarily advantageous, it requires a complex packaging mechanism to ensure the incorporation of the complete set of eight different vRNPs into the progeny virions. In IAV, coordinated genome packaging occurs through terminal packaging signals (TPS) present in all eight vRNAs (4–8). These TPS are located at the 3′ and 5′ termini and span the non-coding regions and additional stretches of the adjacent coding region. IAVs with synonymous mutations in TPS often fail to efficiently package one or multiple vRNAs and therefore produce high numbers of noninfectious viral particles (9–11). The most plausible mechanistic model on how TPS coordinate genome packaging is through the formation of a network of vRNA–vRNA interactions that assembles a supramolecular vRNP complex (12). While a large number of packaging mutants support this model (9–11, 13), specific vRNA–vRNA interactions involving TPS have not yet been identified, leaving the underlying molecular details unresolved (12).

Another model that has recently gained attention is the modulatory role of vRNA–NP interactions in IAV genome packaging. NP serves as the structural scaffold of vRNPs and consists of a head domain, a body domain, and a tail loop (14). Within vRNPs, each NP protomer binds approximately 12 nucleotides (15, 16), and vRNA regions located between such NP-bound sites tend to form secondary structures. NP binds the vRNAs unevenly and without clear sequence or structure specificity (17, 18), possibly through electrostatic interactions between a positively charged RNA-binding groove located between the head and body domains of NP and the negatively charged sugar-phosphate backbone of the vRNAs (14, 19, 20). Interestingly, previous mutagenesis experiments have shown that substitutions near the RNA-binding groove cause a mutant virus to mispackage multiple vRNAs, a phenotype similar to that of viruses with mutated packaging signals (21).

Here, we address the importance of NP charge changes for coordinated genome packaging using a mutagenesis-based approach focusing on acetylated lysine residues inside and outside the RNA-binding groove of NP (22). We find that substitution of single lysine residues with either neutrally charged glutamine (mimicking the acetylated state) or positively charged arginine (mimicking the non-acetylated state) in the context of mutated TPS leads to mispackaging of specific vRNA subsets, depending on the charge state of the amino acid substitution and its location in the RNA-binding groove. Our data provide indirect evidence that vRNA-NP interactions play a critical role in genome packaging. Thus, we postulate that the RNA-binding groove of NP can adopt variable charge states through targeted lysine (de)acetylation that defines specific binding of the TPS and regulate their activity.

## RESULTS

### Single K-to-R or K-to-Q substitutions at positions 184 and 229 in the RNA-binding groove of NP do not affect genome packaging

**Fig 1:**
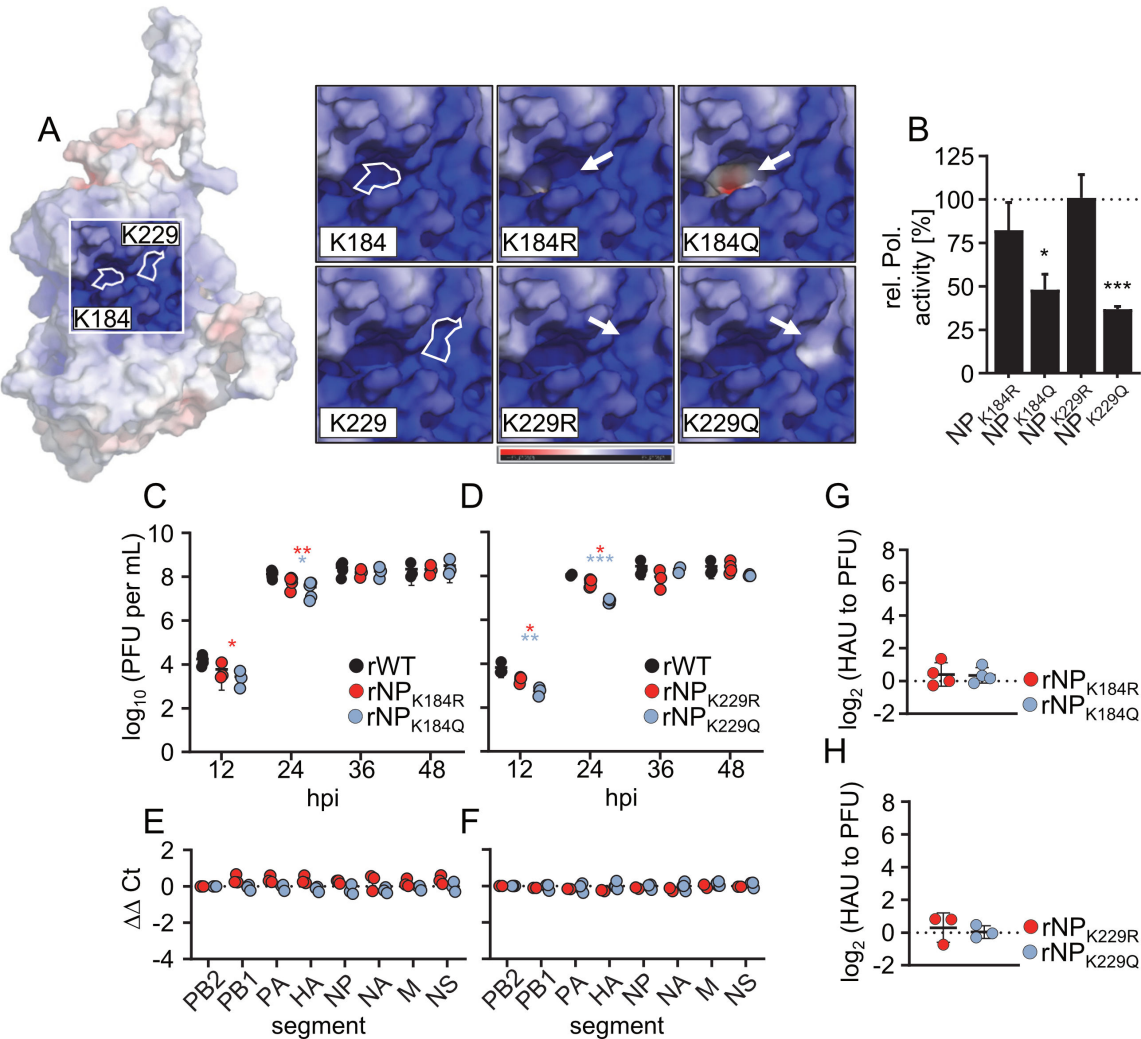
SC35M viruses with a single amino acid substitution at K184 or K229 show no genome-packaging defects. (A) Model of NP showing the relative electrostatic surface potential, with red surfaces representing regions of high electron density and blue surfaces representing regions of low electron density. The insets show surface charges of K184 or K229 with the indicated amino acid exchanges. (B) Polymerase reconstitution assay on transiently transfected HEK293T cells. Plasmids encoding the SC35M polymerase subunits PB2, PB1, and PA were transiently transfected together with plasmids encoding the indicated NP variant (*n* = 3 independent experiments). Data are mean ± SEM. Statistical analysis was performed using Student’s *t*-test. *P < 0.05, ***P < 0.001. (C and D) Multicycle growth kinetics of the indicated mutant viruses compared to those of the wild-type SC35M (rWT). Viral titers were determined at the indicated time points by plaque assay (*n* = 3 to 5 independent experiments). Data are mean ± SD. Statistical analysis was performed using Student’s *t*-test. *P < 0.05, **P < 0.01, ***P < 0.001. (E and F) The amount of the eight genome segments packaged into mutant virions relative to wild-type virions was measured by RT-qPCR at 24 hpi (*n* = 3 independent experiments). Statistical analysis was performed using two-way analysis of variance (ANOVA) with Bonferroni’s multiple comparisons test. (G and H) The relative number of noninfectious particles produced by mutant viruses compared to that of the rWT was determined by hemagglutination assay and plaque assay at 24 hpi. Log_2_-transformed fold changes are shown (*n* = 3 independent experiments). Statistical analysis was performed using one-sample *t*-test.

Some of the previously identified amino acid residues that regulate IAV genome packaging (“NP-packaging code”) (21) are located either within or in close proximity to the postulated RNA-binding groove of NP (Fig. S1A) (14, 23). The RNA-binding groove contains several highly conserved arginine (R) residues, one tyrosine (Y) residue, and two lysine (K) residues 184 and 229 (Fig. S1A) (23). Together, these residues form a positively charged patch located between the NP head and body domain (Fig. S1B), which is thought to interact with the negatively charged viral RNA backbone (14, 16, 19, 20). It was previously shown that during viral infection, K184 and K229 undergo post-translational modifications that remove the positive charge from their side chain, namely, acetylation (for K184 and K229) and ubiquitination (for K184) (22, 24). To study the effect of different charge states of these lysines associated with (de)acetylation, we replaced K184 or K229 with either arginine (R), which preserves the positive charge similar to a non-acetylated lysine, or glutamine (Q), which introduces a neutral charge similar to an acetyl-lysine. *In silico* modeling showed that the K-to-Q substitutions rendered the select sites in the RNA-binding groove less positively charged or even introduced a negative charge, whereas the K-to-R substitutions largely preserved the charge profile of the RNA-binding groove (Fig. 1A). To determine whether these amino acid substitutions affect viral polymerase activity, we reconstituted vRNP complexes of A/seal/Massachusetts/1/1980 (SC35M) using NPs with either K184R, K184Q, K229R, or K229Q substitution. While NP_K184R_ and NP_K229R_ had little to no effect on viral polymerase activity, NP_K184Q_ and NP_K229Q_ reduced viral polymerase activity by two- to threefold (Fig. 1B; Fig. S2A), suggesting that removing positive charges from specific sites of the RNA-binding groove through K-to-Q substitutions affects viral polymerase activity.

To study the effects of these NP substitutions in a viral context, we generated recombinant SC35M viruses with single NP amino acid substitutions, designated rNP_K184R_, rNP_K184Q_, rNP_K229R_, and rNP_K229Q_, and performed multicycle growth kinetics in MDCK II cells. Although viral growth of all mutant viruses was slightly impaired at early time points compared to that of wild-type SC35M (rWT), the endpoint titers at 48 hours post-infection (hpi) were similar (Fig. 1C and D). Consistently, quantification of all eight viral genome segments by RT-qPCR showed that the mutant viruses efficiently packaged the complete viral genome and did not generate more non-infectious virus particles than rWT (Fig. 1E through H). These data demonstrate that single amino acid substitutions at residue K184 or K229 in the RNA-binding groove of NP cause no detectable packaging defect.

### K-to-R or K-to-Q substitutions at positions 184 and 229 of NP have unique effects on genome packaging when combined with different mutated TPS

We have previously shown that a combination of seven amino acid substitutions in the NP head domain and one substitution in the NP body domain (referred to as “NP7-R31G”) does not cause a detectable packaging defect *per se*; however, these NP substitutions impair genome packaging when combined with a single mutated TPS (11). To test whether this scenario also holds true for the K184 and K229 substitutions in the RNA-binding groove of NP, we first generated recombinant SC35M viruses carrying NP_K184R_ or NP_K184Q_ together with synonymous mutations in the 5′ TPS of the PB2 segment (rNP_K184R_PB2_TPS_ and rNP_K184Q_PB2_TPS_), PB1 segment (rNP_K184R_PB1_TPS_ and rNP_K184Q_PB1_TPS_), or PA segment (rNP_K184R_PA_TPS_ and rNP_K184Q_PA_TPS_). Importantly, mutant SC35M viruses with only one mutated TPS do not show a detectable genome-packaging defect (rPB2_TPS_ and rPA_TPS_) or show reduced packaging of only the mutated segment (rPB1_TPS_) (Fig. S2B through G) (11).

**Fig 2:**
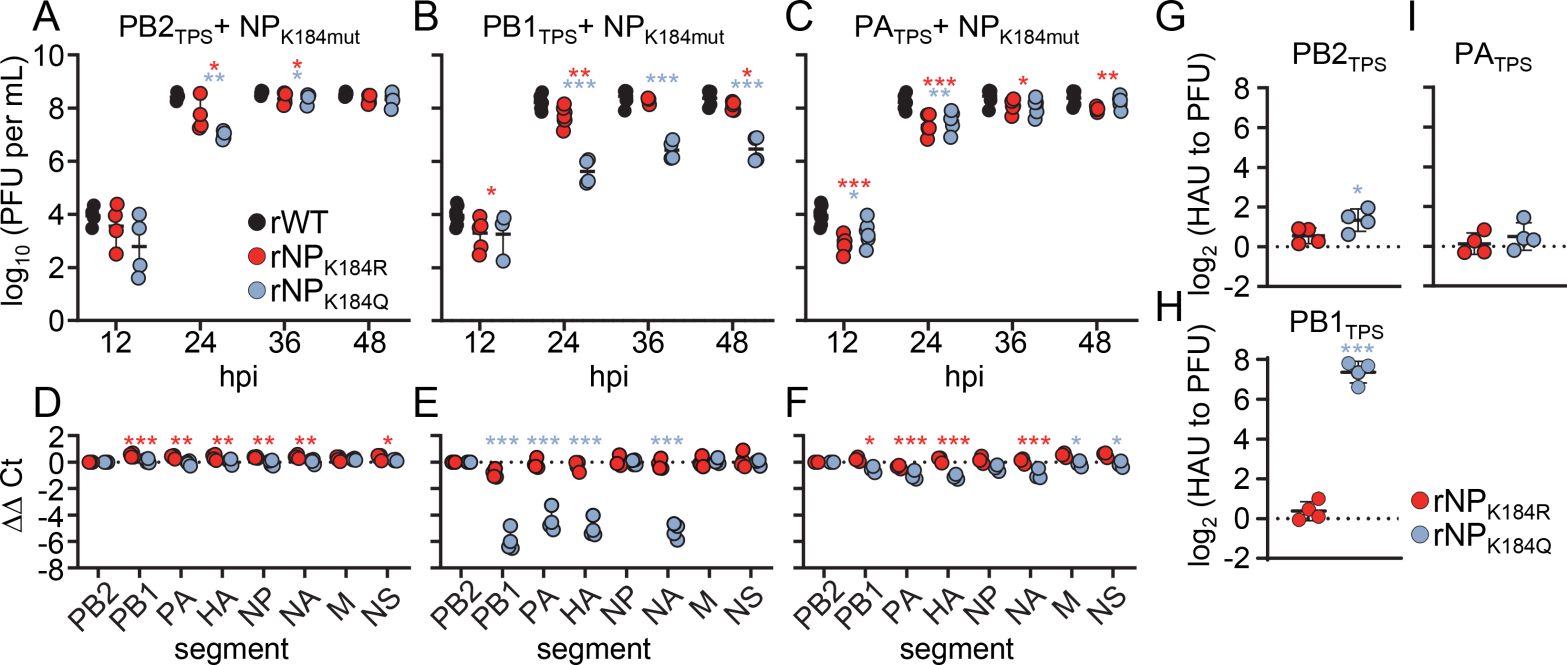
The combination of NP_K184Q_ and the synonymously mutated PB1_TPS_ causes a packaging defect. (A–C) Multicycle growth kinetics of the indicated mutant viruses compared to those of the wild-type SC35M (rWT). Viral titers were determined at the indicated time points by plaque assay (*n* = 3–5 independent experiments). Data are mean ± SD. Statistical analysis was performed using Student’s *t*-test. *P < 0.05, **P < 0.01, ***P < 0.001. (D–F) The amount of the eight genome segments packaged into mutant virions relative to wild-type virions was measured by RT-qPCR at 24 hpi (*n* = 3–4 independent experiments). Statistical analysis was performed using two-way ANOVA with Bonferroni’s multiple comparisons test. (G–I) The relative number of noninfectious particles produced by mutant viruses compared to that of rWT was determined by hemagglutination assay and plaque assay at 24 hpi. Log_2_-transformed fold changes are shown (*n* = 4 independent experiments). Statistical analysis was performed using a one-sample *t*-test.

A combination of the K184Q or K184R substitution with the PB2_TPS_ or PA_TPS_ segment had only a minimal impact on viral growth, as these combinatorial viruses reached similar peak titers as rWT (Fig. 2A and C). Consistently, these mutant viruses did not exhibit inefficient packaging of any genome segment (Fig. 2D and F) and no increased production of non-infectious particles (Fig. 2G and I). In contrast, the rNP_K184Q_PB1_TPS_ mutant showed a pronounced packaging defect, as it replicated to 100-fold lower titers than rWT at 48 hpi (Fig. 2B), packaged the PB1, PA, HA, and NA segments 60- to 20-fold less efficiently (Fig. 2E), and produced 170-fold more non-infectious viral particles (Fig. 2H). However, the rNP_K184R_PB1_TPS_ mutant did not show impaired viral growth at 48 hpi compared to rWT (Fig. 2B), packaged all eight vRNAs efficiently (Fig. 2E), and produced no more non-infectious particles (Fig. 2H), demonstrating that the K184R substitution alleviates the original packaging defect caused by the PB1_TPS_ segment in the context of wild-type NP (Fig. S2F and 11). These results suggest that different charge states of residue K184 in NP affect coordinated genome packaging in the context of the PB1_TPS_ segment but not the PB2_TPS_ or PA_TPS_ segment.

**Fig 3:**
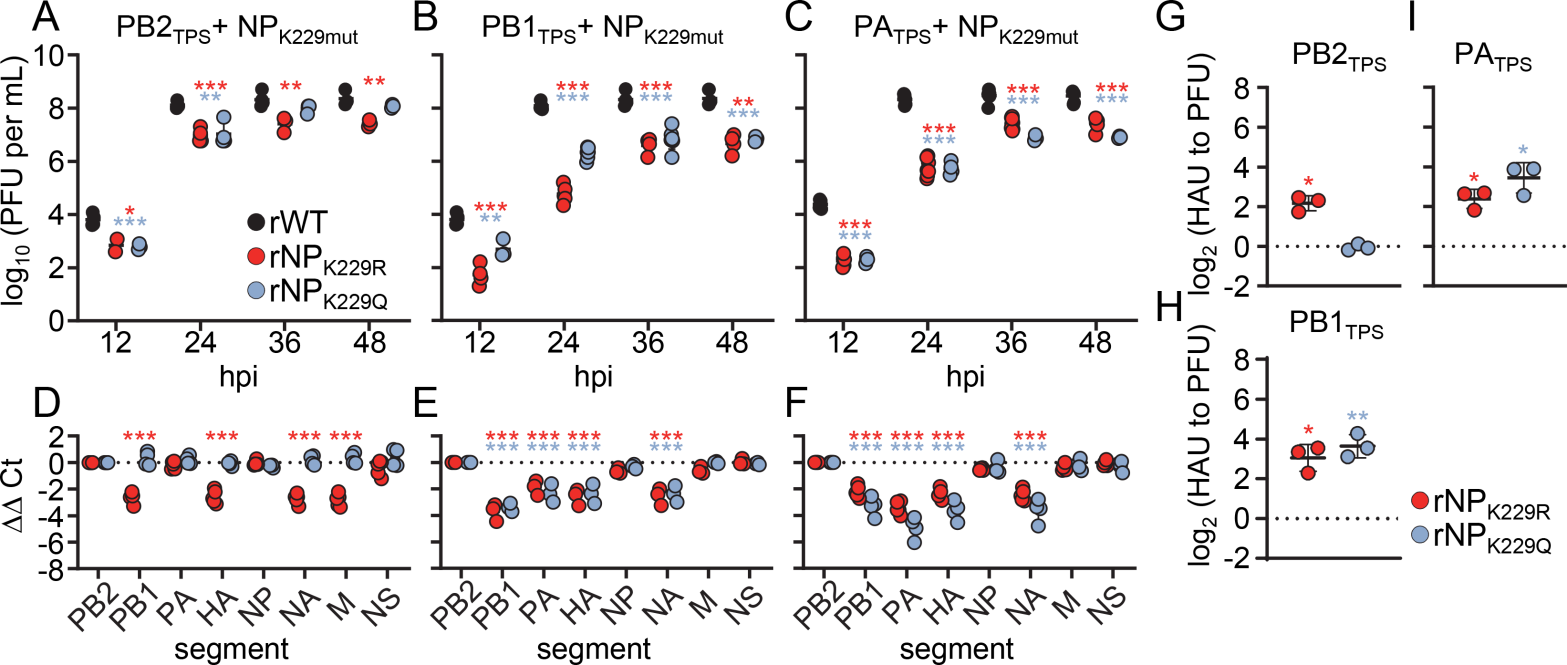
Amino acid substitutions at K229 in combination with synonymously mutated packaging sequences result in genome-packaging defects. (A–C) Multicycle growth kinetics of the indicated mutant viruses compared to those of the wild-type SC35M (rWT). Viral titers were determined at the indicated time points by plaque assay (*n* = 3–5 independent experiments). Data are mean ± SD. Statistical analysis was performed using Student’s *t*-test. *P < 0.05, **P < 0.01, ***P < 0.001. (D–F) The amount of the eight genome segments packaged into mutant virions relative to wild-type virions was measured by RT-qPCR at 24 hpi (*n* = 3–4 independent experiments). Statistical analysis was performed using two-way ANOVA with Bonferroni’s multiple comparisons test. (G–I) The relative number of noninfectious particles produced by mutant viruses compared to that of rWT was determined by hemagglutination assay and plaque assay at 24 hpi. Log_2_-transformed fold changes are shown (*n* = 4 independent experiments). Statistical analysis was performed using one-sample *t*-test.

Next, we generated virus mutants encoding NP_K229R_ or NP_K229Q_ in the context of the PB2_TPS_ segment (rNP_K229R_PB2_TPS_ and rNP_K229Q_PB2_TPS_), PB1_TPS_ segment (rNP_K229R_PB1_TPS_ and rNP_K229Q_PB1_TPS_), or PA_TPS_ segment (rNP_K229R_PA_TPS_ and rNP_K229Q_PA_TPS_). With the exception of rNP_K229Q_PB2_TPS_, viral growth of all combinatorial mutants was attenuated in MDCK II cells compared to that of wild-type SC35M (Fig. 3A through C). The most pronounced growth defect was observed for rNP_K229R_PB1_TPS_ with 60-fold lower infectious particles at 48 hpi (Fig. 3B), followed by rNP_K229Q_PB1_TPS_ (Fig. 3B) and rNP_K229Q_PA_TPS_ with 40-fold lower titers (Fig. 3C), 10-fold reduced titers for rNP_K229R_PA_TPS_ (Fig. 3C) and fivefold lower titers for rNP_K229R_PB2_TPS_ (Fig. 3A). RT-qPCR analysis of the viral particle content revealed that these observed replication defects resulted from failures to efficiently package specific subsets of the eight distinct vRNAs (Fig. 3D through F). While the rNP_K229R_PB2_TPS_ virus showed reduced incorporation of the PB1, HA, NA, and M segments, the rNP_K229R_PB1_TPS_, rNP_K229Q_PB1_TPS_, rNP_K229R_PA_TPS_, and rNP_K229Q_PA_TPS_ viruses all failed to package the same vRNA subset comprising the PB1, PA, HA, and NA segments (Fig. 3D through F). These packaging-deficient virus mutants also produced four- to 12-fold more non-infectious viral particles compared to wild-type SC35M (Fig. 3G through I). These results suggest that different charge states of residue K229 in NP differentially affect coordinated genome packaging in the context of the PB2_TPS_, PB1_TPS_, and PA_TPS_ segments.

### Different neutral amino acids in place of K229 cause similar genome-packaging defects

**Fig 4:**
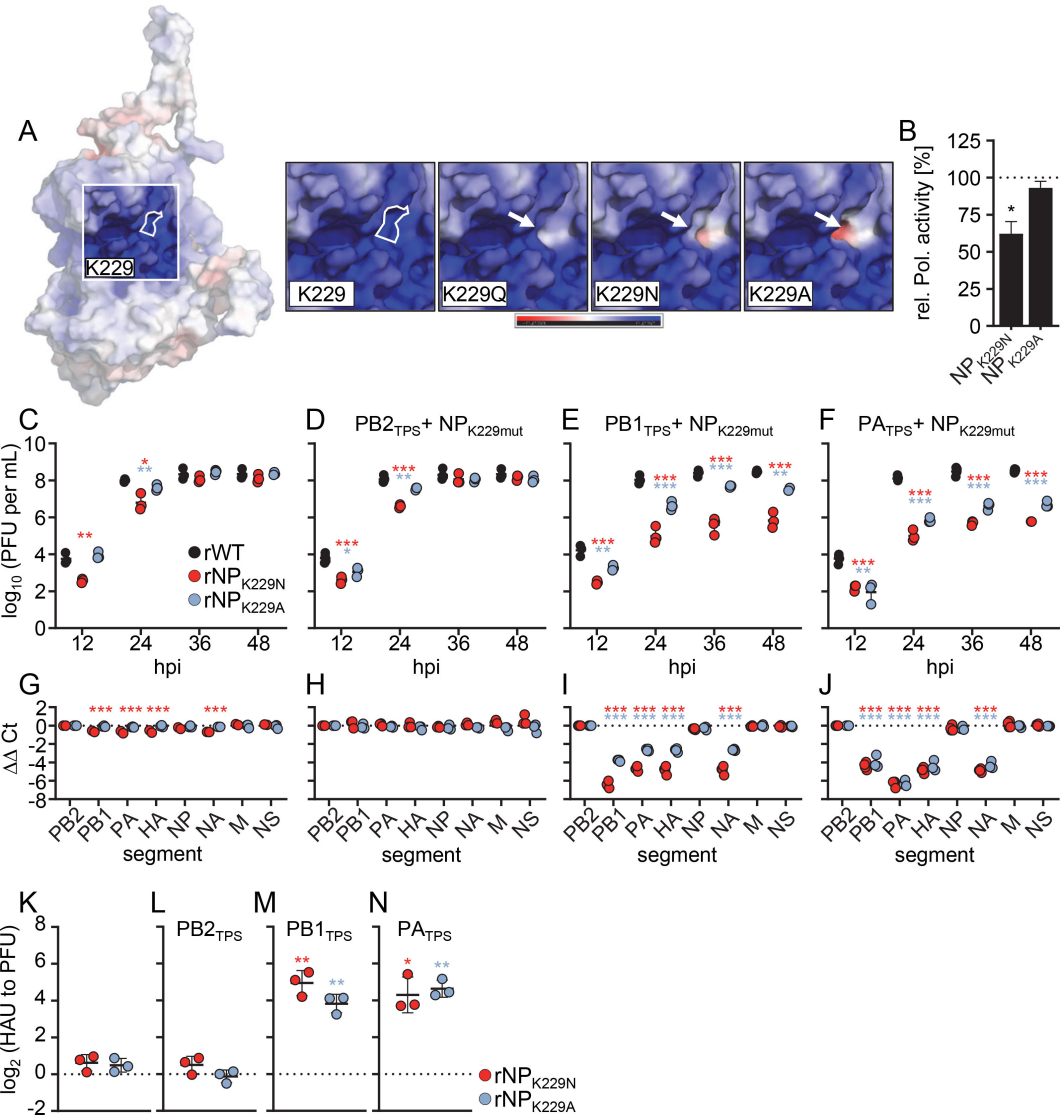
Amino acid substitutions at K229 in combination with synonymously mutated packaging sequences result in genome-packaging defects. (A) Model of NP showing the relative electrostatic surface potential, with red surfaces representing regions of high electron density and blue surfaces representing regions of low electron density. The insets show the surface charge of K229 with the indicated amino acid exchanges. (B) Polymerase reconstitution assay on transiently transfected HEK293T cells. Plasmids encoding the SC35M polymerase subunits PB2, PB1, and PA were transiently transfected together with plasmids encoding the indicated NP variant (*n* = 3 independent experiments). Data are mean ± SEM. Statistical analysis was performed using Student’s *t*-test. *P < 0.05, ***P < 0.001. (C–F) Multicycle growth kinetics of the indicated mutant viruses compared to those of the wild-type SC35M (rWT). Viral titers were determined at the indicated time points by plaque assay (*n* = 3 independent experiments). Data are mean ± SD. Statistical analysis was performed using Student’s *t*-test. *P < 0.05, **P < 0.01, ***P < 0.001. (G–J) The amount of the eight genome segments packaged into mutant virions relative to wild-type virions was measured by RT-qPCR at 24 hpi (*n* = 3 to 4 independent experiments). Statistical analysis was performed using two-way ANOVA with Bonferroni’s multiple comparisons test. (K–N) The relative number of noninfectious particles produced by mutant viruses compared to that of rWT was determined by hemagglutination assay and plaque assay at 24 hpi. Log_2_-transformed fold changes are shown (*n* = 3 independent experiments). Statistical analysis was performed using one-sample *t*-test.

To substantiate that the observed packaging effects were due to the altered charge state of the specific amino acid substitutions and not due to other properties of the amino acid side chain, we replaced K229 with alanine (A) or asparagine (N), which, like glutamine (Q), are neutrally charged. *In silico* modeling showed that the K229N and K229A substitutions locally removed positive charge from the RNA-binding groove like K229Q, and even rendered it slightly more negatively charged (Fig. 4A). Reconstitution of the viral polymerase complexes showed that the K229N substitution reduced the viral polymerase activity by 40% similar to the K229Q substitution, whereas the K229A substitution caused only minor reduction in polymerase activity (Fig. 1B and 4B; Fig. S2A). The virus mutants rNP_K229A_ and rNP_K229N_ replicated to endpoint titers that were comparable to that of wild-type SC35M, efficiently packaged all eight vRNAs, and did not generate more non-infectious particles (Fig. 4C, G and K). The combinatorial virus mutants rNP_K229A_PB2_TPS_ and rNP_K229N_PB2_TPS_ exhibited reduced viral replication properties between 12 and 24 hpi, but reached peak titers that were similar to that of wild-type SC35M (Fig. 4D). These mutant viruses also efficiently packaged all eight genome segments and produced similar amounts of non-infectious particles (Fig. 4H and L). In contrast, rNP_K229A_PB1_TPS_, rNP_K229N_PB1_TPS_, rNP_K229A_PA_TPS_, and rNP_K229N_PA_TPS_ showed pronounced growth defects at all time points and produced more non-infectious particles due to defective genome incorporation (Fig. 4E, F,I,J, M and N). As determined by RT-qPCR analysis, these virus mutants uniformly failed to efficiently package the PB1, PA, HA, and NA segments (Fig. 4I and J). Because the packaging phenotypes of the combinatorial mutants with the K229A or K229N substitution (Fig. 4) were similar to that of the combinatorial mutants with the K229Q substitution (Fig. 3), they are likely the result of the introduction of the neutral charge at this site of the RNA-binding groove.

### K-to-R or K-to-Q substitutions in a flexible loop of NP do not affect genome packaging

**Fig 5:**
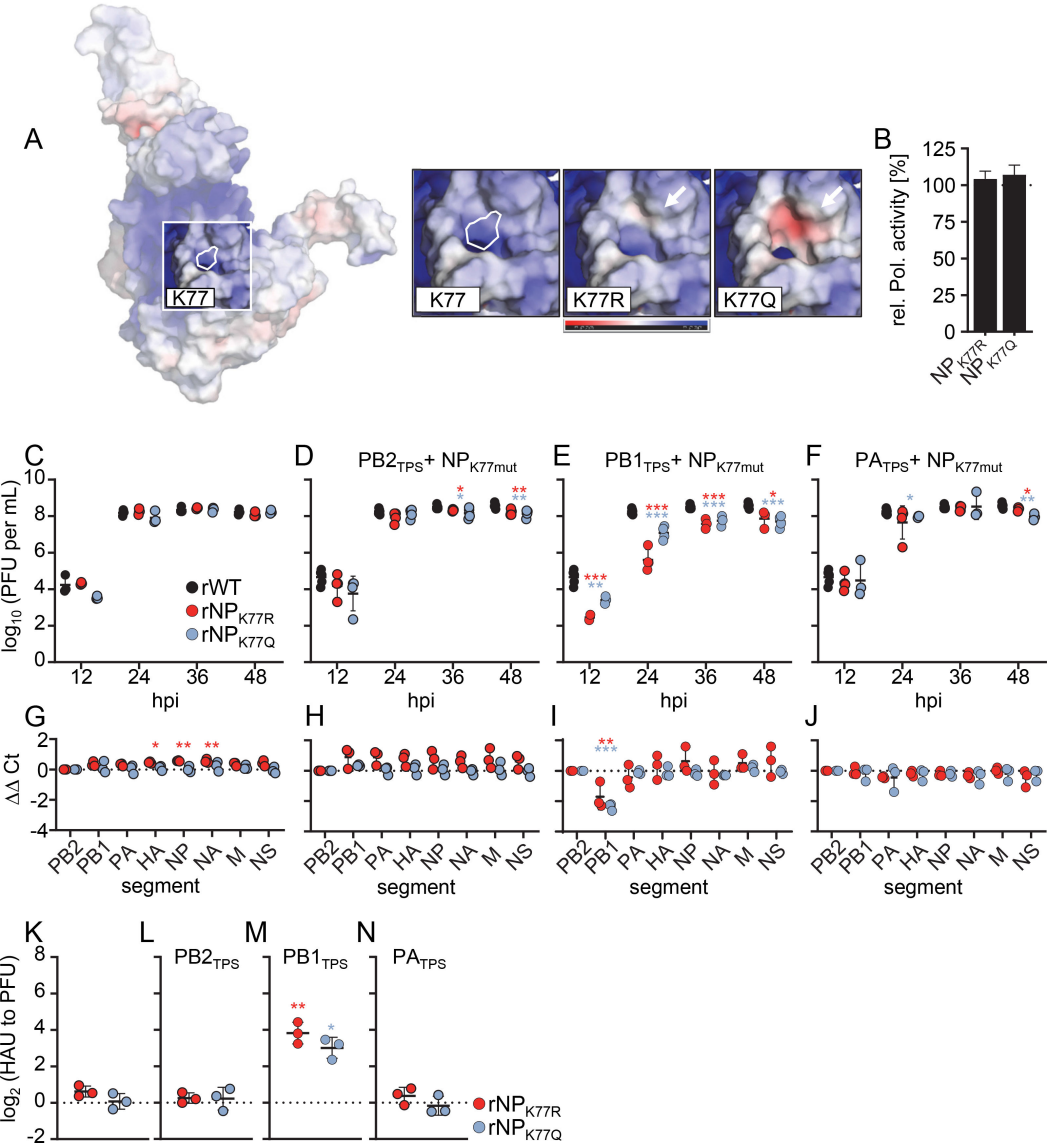
Amino acid substitutions at K77 in the NP body domain do not affect genome packaging. (A) Model of NP showing the relative electrostatic surface potential, with red surfaces representing regions of high electron density and blue surfaces representing regions of low electron density. The insets show the surface charge of K77 with the indicated amino acid exchanges. (B) Polymerase reconstitution assay on transiently transfected HEK293T cells. Plasmids encoding the SC35M polymerase subunits PB2, PB1, and PA were transiently transfected together with plasmids encoding the indicated NP variant (*n* = 3 independent experiments). Data are mean ± SEM. Statistical analysis was performed using Student’s *t*-test. *P < 0.05, ***P < 0.001. (C–F) Multicycle growth kinetics of the indicated mutant viruses compared to those of the wild-type SC35M (rWT). Viral titers were determined at the indicated time points by plaque assay (*n* = 3 independent experiments). Data are mean ± SD. Statistical analysis was performed using Student’s *t*-test. *P < 0.05, **P < 0.01, ***P < 0.001. (G–J) The amount of the eight genome segments packaged into mutant virions relative to wild-type virions was measured by RT-qPCR at 24 hpi (*n* = 3–4 independent experiments). Statistical analysis was performed using two-way ANOVA with Bonferroni’s multiple comparisons test. (K–N) The relative number of noninfectious particles produced by mutant viruses compared to that of rWT was determined by hemagglutination assay and plaque assay at 24 hpi. Log_2_-transformed fold changes are shown (*n* = 3 independent experiments). Statistical analysis was performed using one-sample *t*-test.

Recent data suggest that in addition to the RNA-binding groove, a flexible loop in the NP body domain spanning residues 74–88 may facilitate efficient vRNA binding (20, 25). This loop contains K77, which is acetylated during viral infection similar to K184 and K229 (22). To determine the effect of different charge states of K77, we replaced this residue with either an arginine or a glutamine (Fig. 5A). *In silico* modeling showed that the K77Q substitution introduced a slight negative charge into the loop, whereas the K77R substitution kept this site neutrally charged (Fig. 5A). Polymerase reconstitution demonstrated that both substitutions did not affect the viral polymerase activity (Fig. 5B; Fig. S2A). We then analyzed viral growth properties and the genome content of recombinant viruses harboring the amino acid substitution K77R or K77Q in NP, designated rNP_K77R_ and rNP_K77Q_. Both viral mutants replicated as efficiently as rWT, incorporated all eight vRNAs equally well and produced similar numbers of non-infectious particles (Fig. 5C, G and K). To test the effect of these K77 substitutions in combination with the mutated 5′ TPS of the PB2, PB1, and PA segment, we generated the combinatorial mutants rNP_K77R_PB2_TPS_, rNP_K77Q_PB2_TPS_, rNP_K77R_PB1_TPS_, rNP_K77Q_PB1_TPS_, rNP_K77R_PA_TPS_, and rNP_K77Q_PA_TPS_. Whereas combinatorial mutants carrying the PB2_TPS_ or the PA_TPS_ segment did not show growth defects, inefficient packaging of any genome segment, or increased production of non-infectious particles compared to those of rWT (Fig. 5D, F, H, J, L and N), rNP_K77R_PB1_TPS_ and rNP_K77Q_PB1_TPS_ displayed reduced viral growth, packaged reduced levels of the PB1 segment, and produced 8- to 16-fold more non-infectious particles (Fig. 5E, I and M). However, because the observed packaging defect is associated with the mutated PB1_TPS_ (Fig. S2F and 11), it is unlikely that different charge states of K77 affect genome packaging.

## DISCUSSION

**Fig 6:**
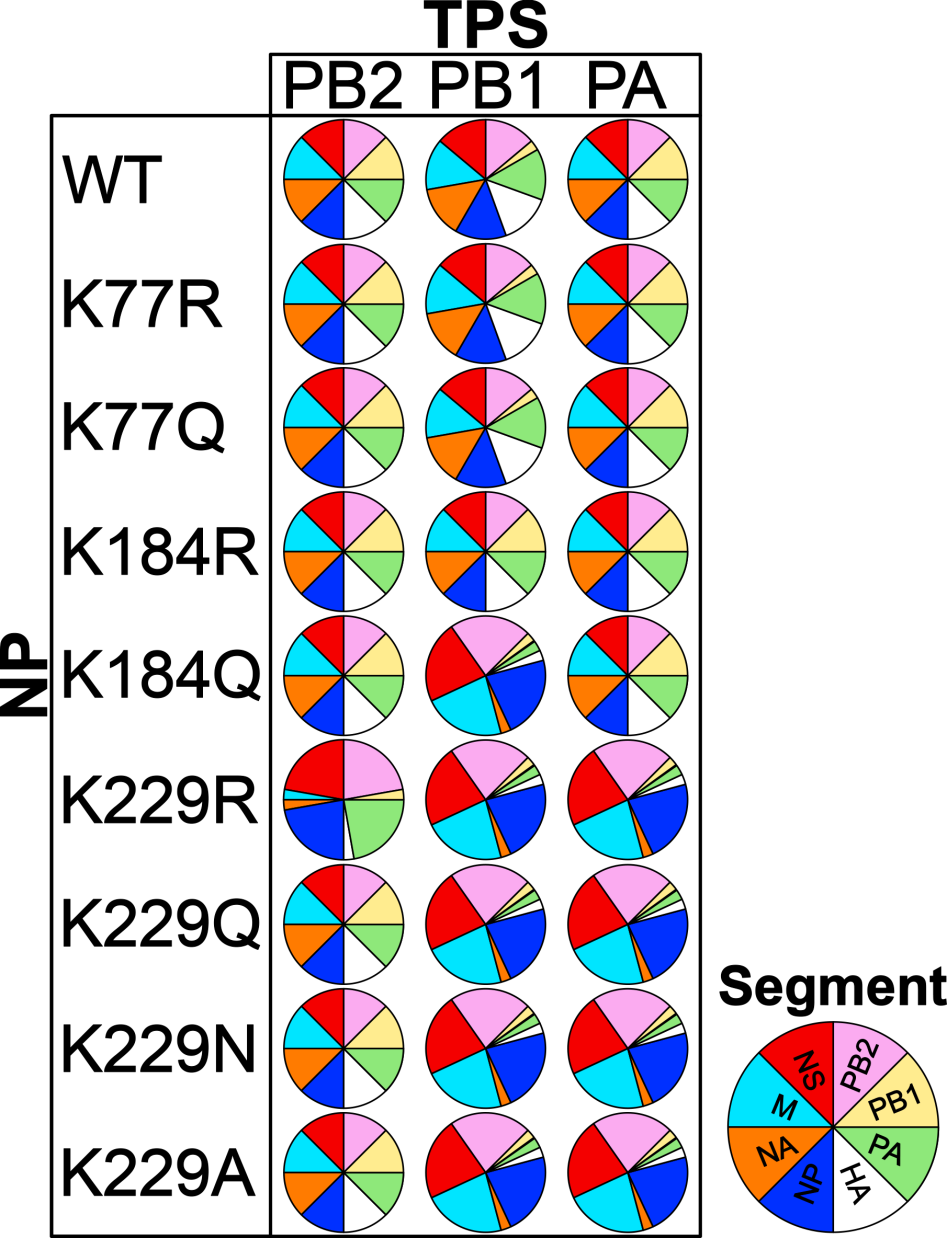
Summary of the observed packaging phenotypes. The figure schematically summarizes the different packaging phenotypes observed by combining mutated terminal packaging signals (TPS) and specific amino acid substitutions at the highly conserved lysine (K) residues 77, 184, and 229 in the RNA-binding groove and the body domain of NP.

We previously identified several lysine residues in the RNA-binding groove of SC35M NP that are acetylated during infection (22). Using K-to-R and K-to-Q substitutions, we provided evidence that (de)acetylation of these lysines controls specific steps in the IAV replication cycle, in particular, genome replication (22). Here, we show that K-to-R and K-to-Q substitutions at distinct sites of the RNA-binding groove of NP, in combination with mutated TPS, differentially affect the packaging of the eight genomic vRNAs as follows: (i) K184 substitutions affect genome packaging only in the context of PB1_TPS_, with K184Q reducing the levels of four specific vRNAs within virions and K184R restoring efficient packaging of the PB1 segment; (ii) the K229R substitution reduces the packaging of specific vRNAs in the context of all three mutated TPS, whereas the K229Q substitution reduces the packaging of specific vRNAs when combined with PB1_TPS_ or PA_TPS_, but not PB2_TPS_; and (iii) neither K77Q nor K77R affect genome packaging in context of any mutated TPS (Fig. 6). This suggests that the presence or absence of a positive charge at different sites of the RNA-binding groove of NP can impair (or promote) the activity of specific TPS, possibly resulting in specific changes in the vRNP-vRNP interaction network. Because the amino acid substitutions in NP alone do not result in detectable packaging defects, we speculate that they alter only a few vRNP-vRNP interactions and that additional disruption of TPS by synonymous mutation is required to impair coordinated genome packaging.

Based on our data, we propose a model in which targeted acetylation (i.e., the removal of a positive charge) or deacetylation (i.e., the restoration of a positive charge) at K184 and K229 helps to establish unique charge distributions in the RNA-binding groove of NP, facilitating electrostatic interactions with TPS that match these charge distributions. These specific TPS–NP interactions may, in turn, induce proper folding of the RNA secondary structure in adjacent unbound TPS regions, thereby triggering vRNA–vRNA interactions required for genome assembly. Alternatively, specific TPS–NP interactions may occur between different vRNPs and directly trigger genome assembly. Our results show that combinatorial mutants with packaging defects fail to incorporate a specific vRNA subset consisting of PB1, PA, HA, and NA (or PB1, HA, NA, and M). Because genome assembly is thought to occur in a stepwise (26–28) and coordinated manner through vRNA–vRNA interactions (4–8), we speculate that genome assembly of these combinatorial mutants is terminated at an early assembly intermediate consisting of the PB2, NP, M, and NS vRNPs, possibly due to impaired interactions with the remaining four vRNPs required for genome assembly (29).

In addition to K184 and K229, K113 and K273 (but not K77) may also be part of a network of lysine residues in the RNA-binding groove that adopt different charge states through (de)acetylation to orchestrate TPS activity (22, 30). Other residues involved may be positively charged arginines within or near the RNA-binding groove, in particular, R74, R156, R174, R175, R195, R199, and R361, which have been implicated in coordinated genome packaging by a previous alanine scan (30), as well as R236, R243, and R246, which are all replaced by lysines in the previously described rNP7 packaging mutant (21). Notably, R150 and R246 undergo methylation during IAV infection (31), potentially representing another post-translational modification that could alter the charge state of the RNA-binding groove and influence TPS activity.

Although we provide the first evidence that interactions of TPS with the RNA-binding groove of NP control IAV genome packaging, it is unclear how different charge states of the RNA-binding groove would lead to the binding of specific TPS. One possibility is that the RNA-binding groove serves as a plastic binding platform with many variable binding sites for different TPS. The specific binding of a given TPS by the cognate binding site in NP may be biased by electrostatic interactions involving locally distributed positive charges in the RNA-binding groove (32). While CLIP experiments could help to identify which TPS are differentially bound by NPs of a wild-type virus versus different NP-packaging mutants, crystal structures of NP in complex with different TPS could shed light on the type of RNA-protein interactions that control the IAV genome packaging.

In conclusion, our study provides evidence that IAV genome packaging is controlled by specific interactions between the RNA-binding groove of NP and the packaging signals of the vRNAs. We propose that these interactions are specified by different charge distributions in the RNA-binding groove corresponding to the different packaging signals. Flexible charge states of the RNA-binding groove may be established by regulated acetylation of multiple lysine residues.

## MATERIALS AND METHODS

### Cell lines

Human embryonic kidney 293T (HEK293T, ATCC CRL-3216) cells and Madin-Darby canine kidney type II (MDCK II, catalog number 00062107; Merck) cells were maintained at 37°C and 5% CO_2_ in Dulbecco’s modified Eagle’s medium (DMEM) supplemented with 10% fetal calf serum, 100 U/ml of penicillin, and 100 mg/mL of streptomycin.

### Plasmids

The pHW2000 plasmids of the H7N7 A/Seal/Massachusetts/1/1980 (SC35M) virus used to generate mutant variants were generated by site-specific mutagenesis. Similarly, pCAGGS expression plasmids coding for NPs with amino acid substitutions at K77, K184, and K229 were generated by site-directed mutagenesis. All primers and plasmids used in this study are listed in Table S1.

### Polymerase reconstitution assay

Sub-confluent HEK293T cells were transfected in 12-well plates with pCAGGS expression plasmids encoding the polymerase subunits PB2, PB1, and PA (each 45 ng) of SC35M together with 150 ng of pCAGGS plasmids coding for the wild-type SC35M NP, NP_K184R_, NP_K184Q_, NP_K229R_, NP_K229Q_, NP_K229A_, or NP_K229N_ using Lipofectamine 2000 (Thermo Fisher Scientific, 11668019) according to the manufacturer’s protocol. The *firefly* luciferase-encoding construct pPolI-FFLuc-RT (25 ng) served as a viral minigenome. Transfection efficiency was determined by co-transfecting 20 ng of the pRL-SV40 plasmid coding for the *Renilla* luciferase. At 24 h posttransfection, cells were lysed, and the *firefly* and *Renilla* luciferase activities were measured using the Dual-Luciferase reporter assay system (Promega).

### Western blot

To determine similar NP expression levels for the reconstituted viral polymerases, HEK293T cells were lysed in Laemmli buffer and subsequently separated by sodium dodecyl sulfate–polyacrylamide gel electrophoresis (SDS-PAGE). Separated protein samples were blotted on a nitrocellulose membrane. Viral NP and host cell HSP90 protein levels were determined using polyclonal rabbit anti-IAV-NP (Gene Tex, GTX125989, 1:1,000) and polyclonal rabbit anti-HSP90 (Santa Cruz Biotechnology, sc-7947; 1:1,000) antibodies. Primary antibodies were detected using peroxidase-conjugated secondary antibodies directed to rabbit IgG (Jackson ImmunoResearch, catalog# 111–035-045, 1:5,000).

### Generation of recombinant viruses

Recombinant SC35M viruses were generated using the eight-plasmid pHW2000 reverse-genetics system as described before (11, 33, 34). Recombinant viruses were plaque purified on MDCK II cells and then used for stock generation. Stock titers were subsequently determined via plaque assay on MDCK II cells. The presence of the introduced mutations was confirmed by Sanger sequencing using segment-specific primer sets listed in Table S1.

### Viral growth kinetics

MDCK II cells were grown to full confluency in six-well plates. The confluent cell layer was washed with PBS and subsequently infected at a multiplicity of infection (MOI) of 0.001 with the indicated virus in infection medium [DMEM containing 0.2% bovine serum albumin (BSA), 100 U/mL of penicillin, and 100 mg/mL of streptomycin]. Viral titers were determined via plaque assay on MDCK II cells at the indicated time points.

### Relative quantification of viral RNA segments per PFU

Confluent MDCK-II cells in six-well plates were infected with recombinant wild-type and mutant SC35M viruses at an MOI of 0.001 PFU per cell. At 24 hpi, cell culture supernatants were collected and cleared by centrifugation for 5  min at 500 × *g*. The amount of infectious particles was quantified using plaque assays on MDCK-II cells. Relative amounts of each packaged vRNA segment from the same virion preparations were quantified by RT-qPCR. Briefly, 200  µL of supernatant was mixed with 500  µL of peqGOLD TriFAST reagent, and RNA was extracted using a Direct-zol RNA MiniPrep kit and eluted in 50  µL of RNase-free water. Subsequently, 4  µL of purified RNA was reverse transcribed with random hexamer primers using a RevertAid first-strand cDNA synthesis kit (Thermo Fisher Scientific, K1621). The cDNA products were diluted 1:25 in Milli-Q water and used for quantitative PCR using a SensiFAST SYBR Hi-ROX kit (Bioline, BIO-92020) with segment-specific primers (Table S1). For this, in 384-well plates (Thermo Fisher Scientific, catalog no. 4309849), 5  µL of the diluted cDNA was mixed with 5 µL of 2× SensiFAST SYBR Hi-ROX mix and 200-nM concentrations of each primer (final concn 160 nM) in a total volume of 12.5 µl. Quantitative PCRs were performed in technical triplicates with a QuantStudio 5 real-time PCR system (Thermo Fisher Scientific). After excluding a packaging defect of the PB2 segment, vRNA levels in the mutants were normalized to those of the wild type and then to the relative PB2 vRNA levels as follows:


$$\Delta \Delta CT=(CT_{WT\_vRNA\_X}-CT_{mut\_vRNA\_X})-(CT_{WT\_vRNA\_PB2}-CT_{mut\_vRNA\_PB2})$$

### Measurements of relative HAU-to-PFU ratios

Confluent MDCK-II cells in six-well plates were washed with PBS and infected with wild-type or mutant SC35M viruses at an MOI of 0.001 PFU per cell in DMEM containing 0.2% BSA and 1% penicillin and streptomycin. Supernatants were harvested at 24 hpi. PFU titers were determined by plaque assay on MDCK-II cells. Hemagglutination titers were determined by HA assay as described previously (49). Briefly, chicken erythrocytes (Labor Merk; E-200) were diluted to 0.75% (vol/vol) in PBS and added to a 1:2 serial dilution of viral supernatant in a 96-round well plate. After 30–60 min of incubation at room temperature, individual wells were monitored for hemagglutination. The HA titer [in hemagglutination units (HAU) per 50 µL] was the lowest virus dilution that produced hemagglutination. The relative log_2_ HAU-to-PFU ratio of the mutant compared to that of the wild-type virus was calculated as follows:


$$log_{2}HAU\text{-}to\text{-}PFU\;ratio=(HAU_{mut}-HAU_{WT})-(log_{2}PFU_{mut}-log_{2}PFU_{WT})$$

### Molecular modeling

The three-dimensional structure of the SC35M NP was generated using the I-Tasser program (https://zhanglab.ccmb.med.umich.edu/I-TASSER/) (35–37) and displayed using the PyMOL software (version 2.5.7). The relative electrostatics were determined using the PyMOL plugin APBS electrostatics 3.0 (Grid Spacing: 0.5; Range: ±5.0).

## Data Availability

The data supporting the findings of this study are available within the paper and its supplemental material. Any further relevant data are available from the corresponding author on reasonable request.

## SUPPLEMENTAL MATERIAL

Fig. S1 jvi.01972-23-s0001.pdf NP structures.

Fig. S2 jvi.01972-23-s0002.pdf Protein expression controls and packaging mutants.

Table S1 jvi.01972-23-s0003.xlsx Overview of the plasmids, primers, and viruses used in this study.

ASM does not own the copyrights to Supplemental Material that may be linked to, or accessed through, an article. The authors have granted ASM a non-exclusive, world-wide license to publish the Supplemental Material files. Please contact the corresponding author directly for reuse.
