# Supplementary material for: Functionality of IAV packaging signals depends on site-specific charges within the viral nucleoprotein
Source: J Virol. 2024 Mar 12;98(4):e01972-23. doi: 10.1128/jvi.01972-23 (PMC11019843; doi:10.1128/jvi.01972-23)
Supplement: Fig. S1 — NP structures. [file jvi.01972-23-s0001.pdf]

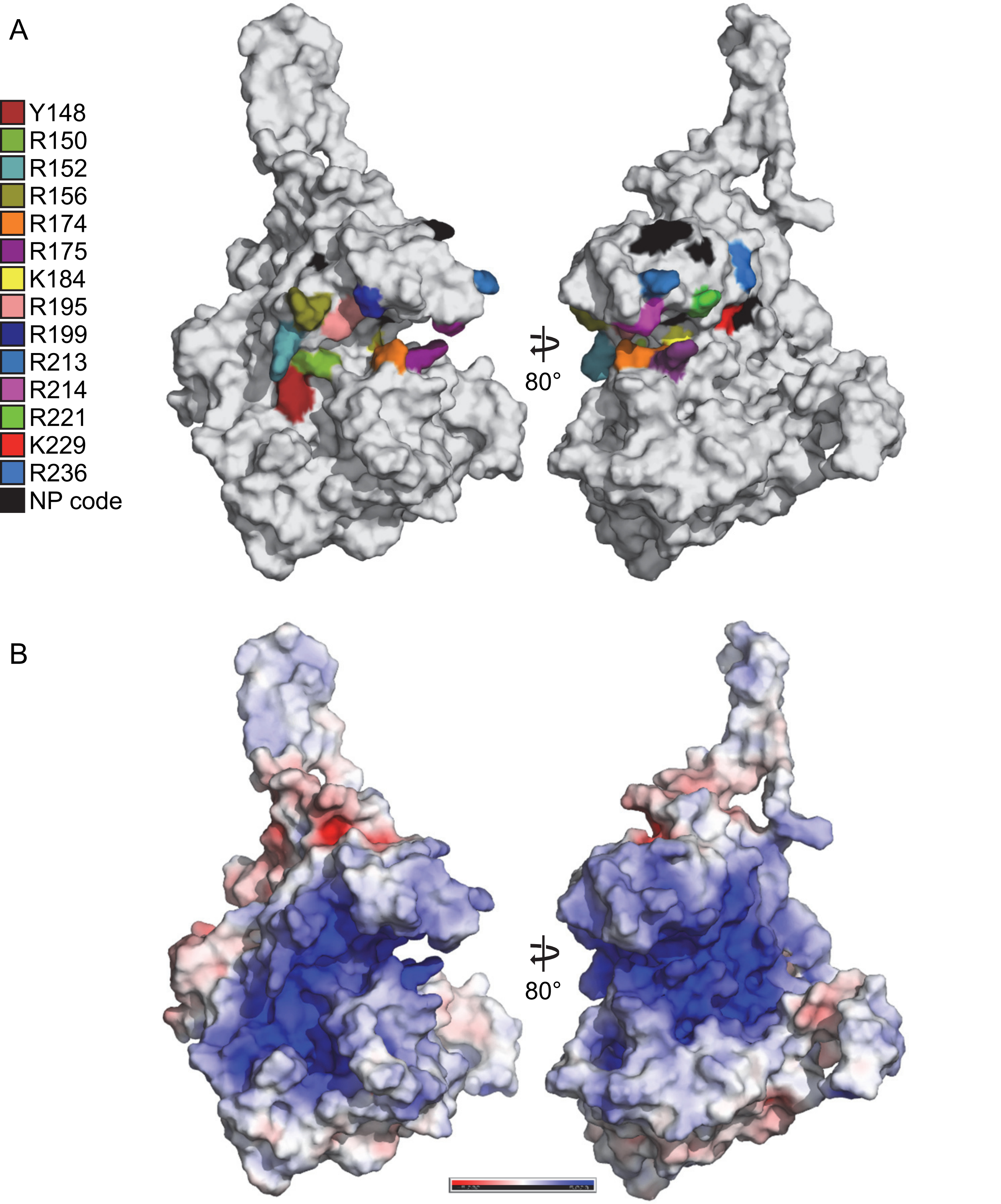

Supplementary Figure 1. A. Schematic representation of the three-dimensional NP structure. Proposed amino acids of the vRNA binding groove are highlighted in different colors. The previously described amino acids that control genome packaging (NP code) are shown in black. Note that R236 is part of the binding groove residues and has been previously identified as NP code residue. **B.** Model of NP showing the relative electrostatic surface potential, with red surfaces representing regions of high electron density and blue surfaces representing regions of low electron density.
