# Supplementary material for: Functionality of IAV packaging signals depends on site-specific charges within the viral nucleoprotein
Source: J Virol. 2024 Mar 12;98(4):e01972-23. doi: 10.1128/jvi.01972-23 (PMC11019843; doi:10.1128/jvi.01972-23)
Supplement: Fig. S2 — Protein expression controls and packaging mutants. [file jvi.01972-23-s0002.pdf]

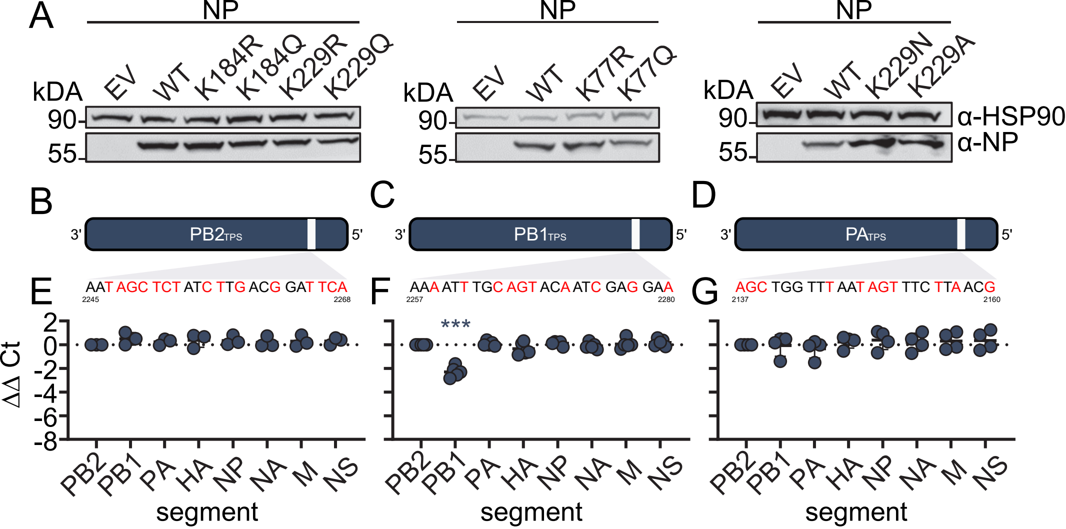

Supplementary Figure 2. A. Expression levels of NP and HSP90 in transfected HEK293T cell lysates were detected by Western blot analysis. **B** to **D.** Schematic representation of the synonymously mutated TPS of the PB2, PB1, and PA segments. Wild-type (black) and synonymously mutated (red) nucleotides are shown and numbered in positive sense. **E** to **G.** The amount of the eight genome segments packaged into mutant virions relative to wild-type virions was measured previously by RT-qPCR at 24 hpi (n= 3 to 4 independent experiments) (11). Statistical analysis was performed using 2way ANOVA with Bonferroni's multiple comparisons test.
